# Mechanisms of Acquired BRAF Inhibitor Resistance in Melanoma: A Systematic Review

**DOI:** 10.3390/cancers12102801

**Published:** 2020-09-29

**Authors:** Ilaria Proietti, Nevena Skroza, Nicoletta Bernardini, Ersilia Tolino, Veronica Balduzzi, Anna Marchesiello, Simone Michelini, Salvatore Volpe, Alessandra Mambrin, Giorgio Mangino, Giovanna Romeo, Patrizia Maddalena, Catherine Rees, Concetta Potenza

**Affiliations:** 1 Dermatology Unit “Daniele Innocenzi”, Department of Medical-Surgical Sciences and Bio-Technologies, Sapienza University of Rome, Fiorini Hospital, Polo Pontino, 04019 Terracina, Italy; nevena.skroza@uniroma1.it (N.S.); nicoletta.bernardini@hotmail.it (N.B.); ersiliatolino@gmail.com (E.T.); veronica.balduzzi@gmail.com (V.B.); anna.marchesiello90@gmail.com (A.M.); simo.mik@hotmail.it (S.M.); salvatore.volpe@uniroma1.it (S.V.); mambrinalessandra@gmail.com (A.M.); patrizia.maddalena@gmail.com (P.M.); concetta.potenza@uniroma1.it (C.P.); 2 Department of Medico-Surgical Sciences and Biotechnologies, Sapienza University of Rome, 00185 Rome, Italy; giorgio.mangino@uniroma1.it (G.M.); Giovanna.romeo@uniroma1.it (G.R.); 3 Department of Infectious, Parasitic and Immune-Mediated Diseases, Istituto Superiore di Sanità, 00185 Rome, Italy; 4 Institute of Molecular Biology and Pathology, Consiglio Nazionale delle Ricerche, 00185 Rome, Italy; 5 Springer Healthcare, Auckland 0627, New Zealand; Catherine.rees@springer.com

**Keywords:** BRAF inhibitors, melanoma, metastasis, microenvironment, resistance, therapy

## Abstract

**Simple Summary:**

Patients with advanced melanoma are often treated with v-raf murine sarcoma viral oncogene homolog B1 (BRAF) inhibitors. Although these agents prolong life, patients inevitably develop resistance and their cancer progresses. This review examines all of the potential ways that melanoma cells develop resistance to BRAF inhibitors. These mechanisms involve genetic and epigenetic changes that activate different signaling pathways, thereby bypassing the effect of BRAF inhibition, but they also involve a change in cell phenotype and the suppression of anticancer immune responses. Currently, BRAF inhibitor resistance can be partially overcome by combining a BRAF inhibitor with a mitogen-activated protein kinase kinase (MEK) inhibitor, but many other combinations are being tested. Eventually, it may be possible to choose the best combination of drugs based on the genetic profile of an individual’s cancer.

**Abstract:**

This systematic review investigated the literature on acquired v-raf murine sarcoma viral oncogene homolog B1 (BRAF) inhibitor resistance in patients with melanoma. We searched MEDLINE for articles on BRAF inhibitor resistance in patients with melanoma published since January 2010 in the following areas: (1) genetic basis of resistance; (2) epigenetic and transcriptomic mechanisms; (3) influence of the immune system on resistance development; and (4) combination therapy to overcome resistance. Common resistance mutations in melanoma are BRAF splice variants, *BRAF* amplification, neuroblastoma RAS viral oncogene homolog (NRAS) mutations and mitogen-activated protein kinase kinase 1/2 (MEK1/2) mutations. Genetic and epigenetic changes reactivate previously blocked mitogen-activated protein kinase (MAPK) pathways, activate alternative signaling pathways, and cause epithelial-to-mesenchymal transition. Once BRAF inhibitor resistance develops, the tumor microenvironment reverts to a low immunogenic state secondary to the induction of programmed cell death ligand-1. Combining a BRAF inhibitor with a MEK inhibitor delays resistance development and increases duration of response. Multiple other combinations based on known mechanisms of resistance are being investigated. BRAF inhibitor-resistant cells develop a range of ‘escape routes’, so multiple different treatment targets will probably be required to overcome resistance. In the future, it may be possible to personalize combination therapy towards the specific resistance pathway in individual patients.

## 1. Introduction

Although melanoma is the least common type of skin cancer, it is the most deadly [1], causing approximately 61,000 deaths per year around the world [2]. An estimated 42–45% of melanomas harbor mutations of the gene for v-Raf murine sarcoma viral oncogene homolog B (BRAF) [3,4], an activating serine/threonine protein kinase in the mitogen-activated protein kinase (MAPK) signaling pathway. In patients with cutaneous melanoma, almost all of these mutations affect codon 600 of exon 15 [3]. The most common mutations are V600E (accounting for ~80% of BRAF mutations), in which a single nucleotide substitution (GTG to GAG) results in valine being substituted for glutamate, and V600K (accounting for ~16% of BRAF mutations), where two nucleotides are affected (GTG to AAG), resulting in valine being substituted for lysine [4]. Other BRAF mutations include V600D and V600R (together accounting for ~3%).

These mutations are oncogenic drivers that cause tumor progression and metastasis, and their discovery led to the development of small molecule inhibitors of BRAF, including vemurafenib, dabrafenib and encorafenib, for the treatment of melanoma [5,6]. Testing for BRAF mutations is now globally recommended in order to choose the most appropriate therapy for patients with stage III or IV melanoma [7,8].

BRAF inhibitors dramatically improved response rate and survival compared with standard chemotherapy in patients with BRAF-mutated melanoma, but these benefits were not durable, and most patients developed progressive disease as a result of resistance development [5]. Consequently, the recommended treatment approach for patients with advanced or metastatic BRAF-mutated melanoma is now a combination of a BRAF inhibitor and a MAPK kinase (MEK) inhibitor [7,8].

Only through thorough understanding of the mechanisms of BRAF inhibitor resistance can we hope to develop strategies for achieving the full therapeutic potential of contemporary treatments in patients with melanoma. Therefore, the aim of the current systematic review was to thoroughly investigate the literature on acquired BRAF inhibitor resistance, in order to identify future potential treatment strategies. Based on what is known about mechanisms of BRAF inhibitor resistance [9], four predefined topics were examined: (1) the genetic basis of resistance; (2) epigenetic and transcriptomic mechanisms of resistance; (3) influence of the immune system on BRAF inhibitor resistance; and (4) the potential of combination therapy to overcome resistance.

## 2. Methods

We undertook a search of the MEDLINE database on May 18, 2020 for any articles on BRAF inhibitor resistance, when used alone or in combination with a MEK inhibitor, in patients with cutaneous melanoma (a full description of the searches is shown in Supplementary Materials) published since the beginning of 2010. Four separate searches were undertaken on each predefined topic. We excluded conference reports/abstracts, news items and case reports, and articles published in languages other than English.

One author (IP) reviewed the search results and chose potential articles based on the title and abstracts. Articles were rejected from the review if they were not specific to acquired BRAF resistance (i.e., the focus was on primary resistance) in cutaneous melanoma (i.e., excluding ocular or mucosal melanoma).

From this group of articles, those most likely to contain pertinent information about BRAF inhibitor resistance mechanisms were extracted for discussion.

## 3. Results

The search identified the following number of articles: (1) 406 on genetic mechanisms, (2) 46 on epigenetic mechanisms; (3) 82 on immune mechanisms; and (4) 499 on overcoming resistance with combination therapy.

A more in-depth review of the articles meant that additional articles were excluded as being either not relevant or not an included article type, and some articles were reclassified into another topic section. The final number of articles included in each section were: (1) 106 articles on genetic mechanisms; (2) 61 articles on epigenetic or transcriptomic mechanisms; (3) 23 articles on immune mechanisms; and (4) 189 articles on overcoming resistance. There was some overlap between sections as some articles were relevant to more than one topic.

### 3.1. Genetic Mechanisms of Resistance

Advances in genetic analytical techniques, such as next generation sequencing (NGS) and clustered regularly interspaced short palindromic repeats (CRISPR) have considerably expanded our knowledge of the genetic changes involved in BRAF inhibitor resistance, and raise the possibility of incorporating mutational information into predictive or prognostic models [10,11,12].

A range of genetic mutations have been identified as causing acquired BRAF resistance (recently reviewed by Tian and Guo and summarized in Table 1) [11,13,14,15,16,17,18,19,20,21,22,23,24,25,26,27,28,29,30,31,32,33,34,35,36,37,38,39,40,41,42,43,44,45,46,47,48,49,50,51,52,53,54,55,56,57,58,59,60,61,62,63,64,65,66,67,68,69,70]. The most common mutations are BRAF splice variants, BRAF amplification, neuroblastoma RAS viral oncogene homolog (NRAS) mutations and MEK1/2 mutations [51,61,71,72], and these appear to be associated with different disease phenotypes. For example, brain metastases appear to be associated with NRAS mutations whereas hepatic progression is associated with MEK1/2 mutations [72]. Longitudinal assessments indicate that patients tend to accumulate resistance-related mutational changes over time [72]. However, the rate of resistance development does not appear to be related to the antitumor activity of the BRAF inhibitor (i.e., the speed at which the BRAF inhibitor kills treatment-sensitive cells has no bearing on the speed at which resistant clones develop) [73].

Splice variants of BRAF mediate resistance by affecting BRAF dimerization. In cells with wild-type BRAF, activation by RAS leads to the formation of homodimers (BRAF-BRAF) or heterodimers with CRAF (BRAF-CRAF), whereas cells with V600E mutations do not form dimers and activate MEK via monomeric BRAF. BRAF inhibitors are ineffective in melanoma with wild-type BRAF because the homo- and heterodimers retain their signaling capacity, whereas these inhibitors block the action of monomeric BRAF. Splice variants of BRAF V600E are also able to form dimers and therefore to activate MEK in the presence of BRAF inhibitors [50,74].

**Figure 1:**
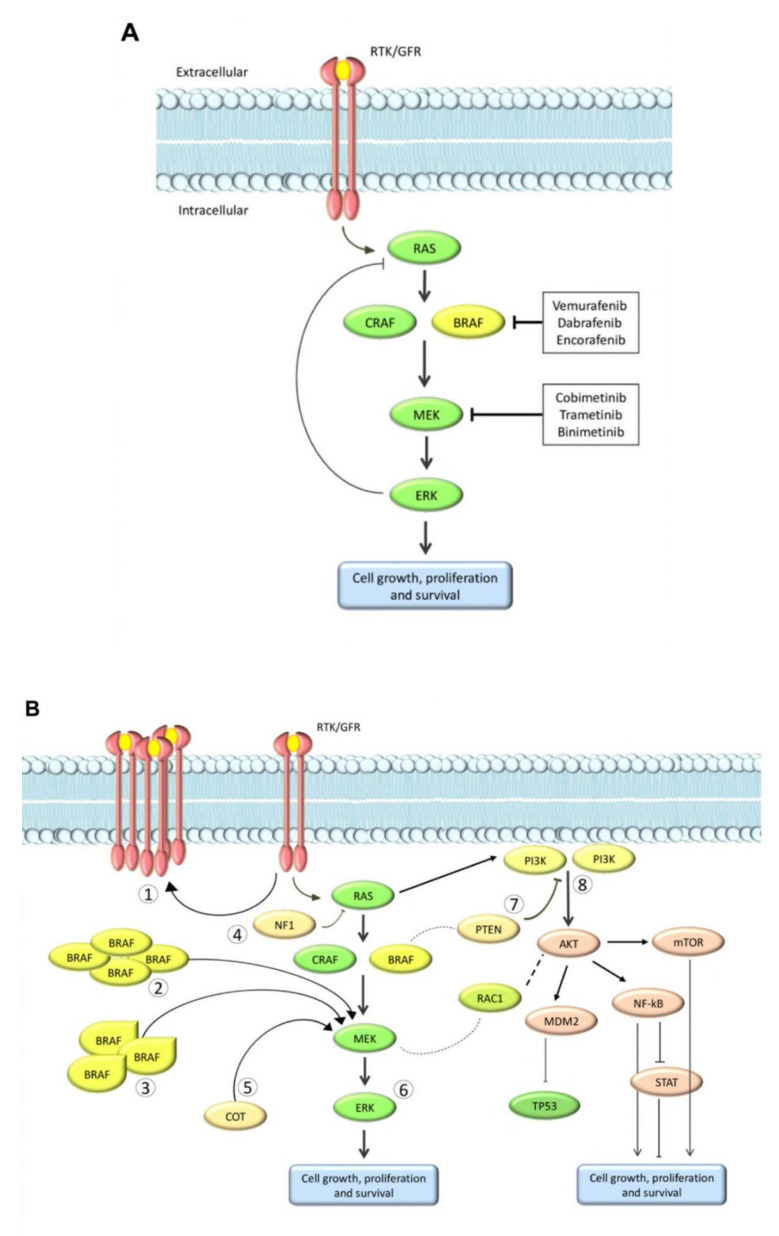
The MAPK signaling pathway (**A**) in a BRAF inhibitor-sensitive cell showing sites of action of BRAF inhibitors and MEK inhibitors, and (**B**) after BRAF inhibitor resistance development [78]. Mechanisms of resistance are numbered: (1) upregulation of RTK; (2) BRAF amplification; (3) BRAF alternative splicing; (4) loss of NF1; (6) ERK activation; (7) loss of PTEN; and (8) activation of alternative signaling pathways. BRAF, v-Raf murine sarcoma viral oncogene homolog B; ERK, extracellular signal-regulated kinase; GFR, growth factor receptor; mTOR, mammalian target of rapamycin; NF1, neurofibromin 1; PTEN, phosphatase and tensin homolog; RTK, receptor tyrosine kinase. From Tanda et al. Frontiers in Molecular Biosciences 2020, 7, 154 [78]. © 2020 Tanda, Vanni, Boutros, Andreotti, Bruno, Ghiorzo and Spagnolo.

Genetic changes to key molecules in the NRAS/BRAF/MEK pathway lead to reactivation of the previously blocked MAPK pathways or activation of alternative signaling pathways, such as the phosphatidylinositol 3-kinase (PI3K)/protein kinase B (AKT) pathway (Figure 1) [11,36,75,76,77,78]. In some patients with BRAF inhibitor resistance, the activation of PI3K/AKT is driven by loss of phosphatase and tensin homolog (PTEN) expression [79].

The genetic changes also contribute to the increased cytoprotective autophagy seen in BRAF inhibitor-resistant melanoma cells [80,81,82,83], allowing tumor cell proliferation to continue unchecked. The enhanced autophagic-lysosomal activity of BRAF-resistant melanoma cells exacerbates adenosine triphosphate (ATP) secretion, which in turn increases melanoma cell invasion [82].

However, some mutations are independent of downstream pathways. Overexpressed genes in BRAF inhibitor-resistant cells are often associated with growth factors and their receptors, cell adhesion molecules and extracellular matrix binding [84]. Common mutations involve effects on receptor tyrosine kinases (RTKs), such as epidermal growth factor receptor (EGFR), platelet-derived growth factor receptor (PDGFR), hepatocyte growth factor (HGF), or insulin-like growth factor (IGF) receptor, which in turn activate parallel pathways [11,19,20,25,29,58,59,84,85,86]. Research has shown extensive redundancy in RTK-mediated signaling pathways, whereby a broad range of widely expressed RTKs are upregulated in cells with BRAF inhibitor resistance [86]. These changes are mediated post-translationally via the inhibition of proteolytic ‘shedding’ of cell surface receptors [87]. This shedding is a normal part of the negative feedback loop that limits intracellular signaling, but is blocked by BRAF inhibitors. As a result, there is an increase in cell surface receptor levels in the tumor during treatment, causing activation or enhancement of alternative signaling pathways.

**Figure 2:**
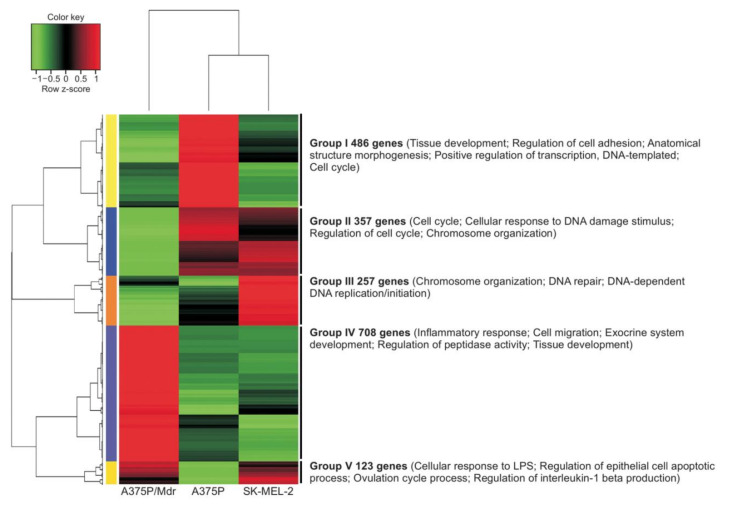
Heat map analysis of genes differentially expressed in cells with BRAF inhibitor sensitivity (A375P), acquired BRAF inhibitor resistance (A375P/Mdr) and innate BRAF inhibitor resistance (SK-MEL-2) [88]. SK-MEL-2 cells have wild-type BRAF and are resistant to BRAF inhibitors because these agents lack activity against wild-type BRAF. From Ahn et al. Biomol 2019, 27, 302–310 [88]. Copyright ©2019, The Korean Society of Applied Pharmacology.

Transcriptomic analysis showed that, compared with BRAF inhibitor-sensitive cells, those with acquired resistance had differences in 887 upregulated genes and 1014 downregulated genes [88]. Upregulated genes were mainly Group IV genes involved in inflammatory response, cell migration, exocrine system development, regulation of peptidase activity and tissue development, or Group V genes involved in cellular response to lipopolysaccharide, regulation of epithelial cell apoptosis, processes involved in the ovulation cycle, and regulation of interleukin (IL)-1β production (Figure 2) [88].

The contribution to resistance of genetic alterations in cell cycle regulators is an interesting finding. One such change is upregulation of the cell cycle genes CDK6 and CCND1 [89]. This appears to open up the possibility of using CDK inhibitors to overcome resistance; preclinical data indicate that adding palbociclib to treatment with BRAF inhibitors and/or MEK inhibitors prevented resistance development in treatment-naïve melanoma cells and animal models, but did not overcome resistance in cells and animals with acquired BRAF inhibitor resistance [90].

Other genetic changes implicated in the development of vemurafenib resistance are loss of genes that encode NF1 (a negative regulator of RAS) and CUL3 (a key protein in the ubiquitin ligase complex) [27,91,92]. Loss of CUL3 is associated with increased RAC1 activity [91]. RAC1 is a member of Rho family of GTPases that regulate actin dynamics, cytoskeleton organization, and cell motility [93]. Rho GTPases regulate gene transcription via the downstream co-activator YAP1 [45,94]. Inhibition of actin remodeling, possibly by inhibiting YAP1, has been suggested as a potential target for overcoming BRAF inhibitor resistance [94,95]. Actin is not the only cytoskeleton protein implicated in BRAF inhibitor resistance: differentially regulated genes coding for microtubules and the intermediate filament nestin are also involved [96,97].

The net effect of these genetic alterations is a shift in the phenotype of BRAF inhibitor-resistant melanoma cells, resulting in an epithelial-to-mesenchymal transition (EMT) [97,98,99], characterized by changes in cell–cell adhesion, cell-matrix adhesion, cellular polarity, and the cytoskeleton [100]. As well as being mediated by the genetic changes within the cell, this EMT shift is also stimulated by nearby fibroblasts in the tumoral stroma [101], which secrete growth factors (such as HGF) that strongly activate the MAPK cascade [102].

### 3.2. Epigenetic and Transcriptomic Mechanisms

Plasticity of the melanoma cell phenotype is often driven by changes in the tumor microenvironment, such as hypoxia, pH, and nutrient supply [103,104]. BRAF inhibitor treatment is also associated with a change in the metabolic profile of cells, with a shift towards more mitochondrial respiration and the formation of reactive oxygen species [103,105,106,107,108,109,110].

These changes in the cellular metabolic profile and in the tumor microenvironment affect the activity of histone-modifying enzymes, including histone methyltransferase, histone demethylase, and histone deacetylase (HDAC) [111]. These changes modulate transcription by modifying chromatin structure. For example, the increased oxidative metabolism in BRAF inhibitor-treated cells causes a shift from glucose to glutamine metabolism [103], with increased glutamine catabolism. Low glutamine levels in the core of a melanoma induce histone hypermethylation and BRAF inhibitor resistance [112]. BRAF inhibitor-resistant cells show increased expression of KDM5B, a histone demethylase enzyme [113].

Downregulation of a range of HDAC genes have been reported to be associated with BRAF inhibitor resistance, including SIRT2 (encoding for sirtuin 2) [114], SIRT6 (encoding for sirtuin 6) [115], and HAT1 (encoding for histone acetyltransferase 1) [116]. On the other hand, HDAC8 is upregulated in BRAF inhibitor-resistant cells [117].

Noncoding portions of DNA are also implicated in the development of resistance, particularly loci that are involved in transcription factor recruitment and occupancy [118]. Long non-coding RNA (lncRNA) loci are also implicated; transcriptional activation of one such lncRNA, EMICERI, activates a neighboring gene that confers resistance to BRAF inhibitors [119].

**Figure 3:**
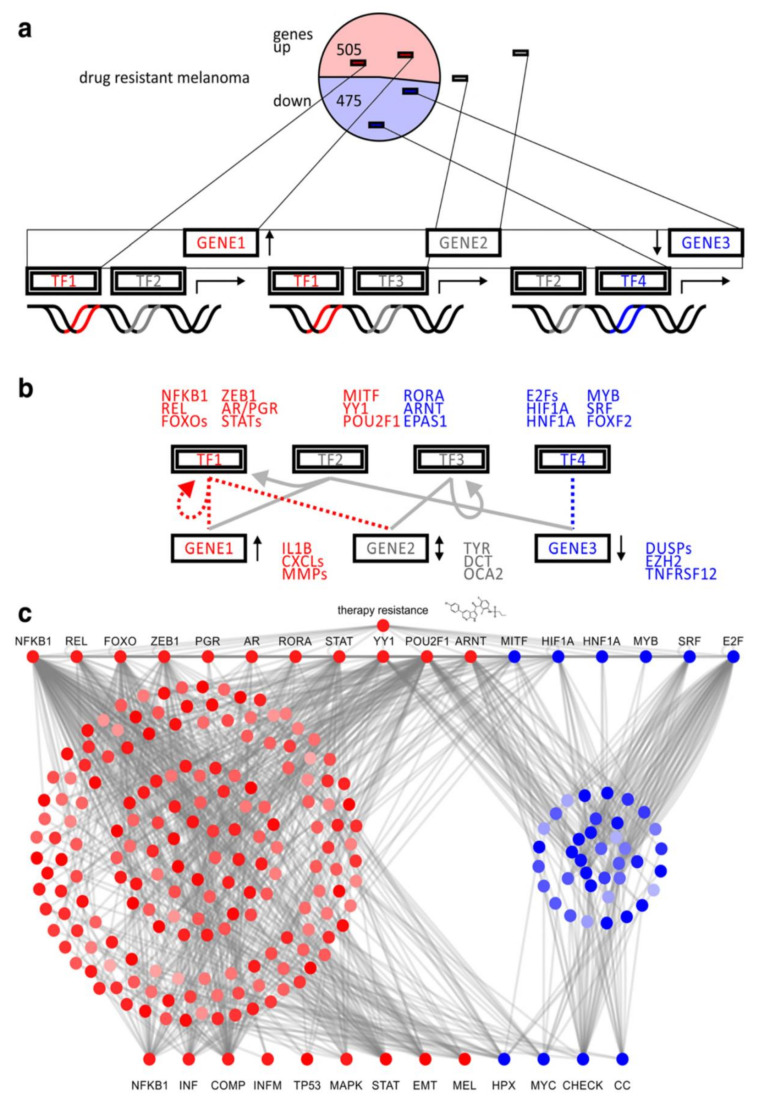
Transcription factor motif analysis of BRAF inhibitor resistance in cellular models of malignant melanoma [141]. Red indicates transcription factors relevant to upregulated genes and blue indicates transcription factors relevant to downregulated genes. (**a**) Schematic representation of differentially expressed genes in a drug resistance model and transcription factor motifs associated with regulated target genes. Upregulated factors are depicted in red and downregulated factors in blue. (**b**) Hierarchical transcription factor network with master regulators on top and downstream targets at bottom. Sets of transcription factor target genes are identified in enrichment analysis based on sequence motifs. (**c**) Hierarchical network model illustrates how therapy resistance in cancer selects for specific transcriptional master regulators to rewire target genes in effector pathways in a concerted fashion. From Zecena et al. BMC Syst Biol 2018, 12, 33 [141]. Copyright © 2018, Zecena, Tveit, Wang, Farhat, Panchal, Liu, Singh, Sanghera, Bainiwal, Teo, Meyskens, Liu-Smith, and Filipp.

BRAF inhibitor resistance involves a range of transcription factors (Table 2) [14,16,22,28,45,46,48,52,58,66,81,89,97,117,120,121,122,123,124,125,126,127,128,129,130,131,132,133,134,135,136,137,138,139,140]. A transcriptomic analysis of BRAF inhibitor-resistant melanoma cells showed that some transcription factors were upregulated whereas others were downregulated [141]. These researchers highlighted a set of transcriptional ‘master regulators’ including STAT, FOXO, ZEB1 (upregulated) and MITF, HIF1A and MYB (downregulated) that control a range of effector pathways (Figure 3). One of the key processes regulated by these transcription factors is ErbB3 phosphorylation, which leads to PI3K/AKT activation [52]. Downstream effects of these transcription-related events include inhibition of apoptosis [142] and the EMT phenotypic change seen in BRAF inhibitor-resistant cells [99,135].

Resistance is also mediated at a post-transcriptional level through effects on translation mediated by RNA binding proteins (e.g., human antigen R and 4E-BP) [143,144], translation initiation complexes (e.g., eIF4F or eIF4E) [144,145], micro-RNAs (Table 2) [59,146,147,148,149,150,151,152,153,154], and by modifications in wobble tRNA [155]. Some of these micro-RNAs (e.g., miR-199b-5p) are regulators of cell-cell signaling via VEGF and HIF-1α, while others (e.g., miR-4488 and miR-4443) are involved in autophagy regulation [149].

### 3.3. Immune Mechanisms

The genetic and epigenetic changes associated with BRAF inhibitor treatment affect the interaction between the melanoma tumor and the immune system in various ways. Untreated melanoma is not particularly immunogenic, but treatment with BRAF inhibitors causes a transient increase in antitumor immunogenicity, with recruitment of T-cells and natural killer (NK) cells and a reduction in regulatory T-cells (Tregs) [156].

Treated patients may develop immune-related adverse events (e.g., arthralgia, immune-related skin reactions), which can be a marker of response to BRAF inhibitors [157,158,159]. However, once resistance to BRAF inhibitors develops, the tumor microenvironment reverts to its low immunogenic state, with fewer infiltrating T-cells and NK cells [9,160], more double-negative T-cells [9,161], and restoration of myeloid-derived suppressor cells [162]. Moreover, the T-cells and NK cells that are present are functionally impaired compared with BRAF inhibitor-sensitive tumors [160], such that cytotoxic T-lymphocytes and NK cells are less effective at recognizing and killing BRAF inhibitor-resistant cells [163,164].

In the tumor cell, this reversion to a low immunogenic state is caused by induced expression of programmed cell death ligand-1 (PD-L1) [165,166,167,168,169], increased expression of the immunoregulatory protein Galactin-1 [170], and increased expression of CD47, an immunoregulatory cell marker, on tumor cells, leading to reduced killing by cytotoxic T-cells and macrophages [133]. BRAF inhibitor-resistant tumor cells also show reduced expression of target antigens [164], and increased production of IL-10 [33]. They also show upregulation of the IGF receptor, which sensitizes BRAF inhibitor-resistant cells to the effects of cytotoxic T-lymphocytes by increasing the cellular uptake of granzyme B [171].

Another change which may have therapeutic implications is the restoration of carcinoembryonic antigen-related cell adhesion molecule 1 (CEACAM1) expression. CEACAM1 is an intercellular adhesion molecule that regulates cell proliferation, cellular energetics, and inflammation in cancer cells, including melanoma, and has a role in regulating the immune cells in the tumor microenvironment [172,173]. CEACAM1 is downregulated prior to resistance development [174], suggesting that it may be a future target for the treatment of BRAF inhibitor-resistant melanoma.

In addition to the changes within the tumor cell, secreted soluble factors originating from macrophages in the tumor microenvironment contribute to resistance, including tumor necrosis factor-α (TNF-α) and vascular endothelial growth factor (VEGF), which contribute to tumor cell growth and invasion, and further infiltration of macrophages and T-lymphocytes into the tumor microenvironment [175,176].

### 3.4. Overcoming Resistance via Treatment Combinations

The standard of care for BRAF-mutated melanoma is now the combination of BRAF inhibitors and MEK inhibitors [7,8], as there is considerable high-quality evidence from randomized comparative studies that this approach prolongs progression-free survival (PFS) and overall survival (OS) compared with BRAF inhibitor monotherapy [177,178,179,180,181,182,183,184,185,186,187]. Some patients are able to achieve a durable response lasting ≥ 5 years with such combinations [183,186].

MEK inhibitors block the MEK/ERK signaling pathway that is activated in cells during treatment with BRAF inhibitors, thereby delaying the development of resistance and increasing the duration of response [188,189]. MEK inhibitors also suppress the production of PD-L1 [167], and so help to suppress immune activation that occurs during BRAF inhibition. The most extensively researched BRAF inhibitor + MEK inhibitor combinations are vemurafenib + cobimetinib [178], dabrafenib + trametinib [184,185,187,190,191,192], and encorafenib + binimetinib [179,180].

While there are no head-to-head comparisons of these combinations, some differences do exist. Encorafenib has the highest paradox index of the three BRAF inhibitors [193]. This index is a ratio of the EC_80_ for ERK activation relative to the IC_80_ of BRAF inhibitor-resistant cell growth, and provides a measure of the therapeutic window for antitumor activity before paradoxical ERK activation [193]. The paradox index of encorafenib is 50, compared with 10 for dabrafenib and 5.5 for vemurafenib. This difference may help to explain the higher efficacy of encorafenib monotherapy compared with vemurafenib monotherapy in the COLUMBUS study [180].

Although these combinations have not been directly compared, the combination of encorafenib + binimetinib was associated with numerically longer OS and PFS in the COLUMBUS study than was seen with vemurafenib + cobimetinib in the coBRIM study or dabrafenib + trametinib in the COMBI-v study [181]. While the overall rate of adverse events with each combination was similar, encorafenib + binimetinib was associated with a lower incidence of pyrexia compared with dabrafenib + trametinib and of photosensitivity compared with vemurafenib + cobimetinib [181].

Once patients progress after BRAF inhibitor + MEK inhibitor treatment, the recommended second-line approach is immunotherapy with an immune checkpoint inhibitor [7]. Some patients who progress on BRAF inhibitor treatment may benefit from a rechallenge with BRAF inhibitor + MEK inhibitor therapy [192].

Preliminary (phase Ib and II) clinical trials have been conducted using triple therapy with a BRAF inhibitor + MEK inhibitor + PD-L1 inhibitor in mostly BRAF inhibitor-naïve patients, with high rates of response (63–73%) [194,195,196]. In the comparative phase II study, triplet therapy prolonged PFS compared with the BRAF inhibitor + MEK inhibitor doublet combination (median 16.0 vs. 10.3 months; p = 0.043), but was associated with more than two times the rate of grade 3 or 4 adverse events (58.3% vs. 26.7%) [194].

The diversity of genetic changes driving resistance, and the inter- and intra-individual heterogeneity of resistance-associated mutations complicates treatment of BRAF inhibitor-resistant melanoma [130,197,198]. One potential approach is to silence the expression of specific genes using small interfering RNA (siRNA) [199]. However, the clinical application of this approach is hampered by difficulties in delivering siRNA into the cytoplasm of tumor cells without the use of viral vectors, which can cause mutation, immune activation, or inflammation [200]. To the best of our knowledge, none of the non-viral vectors for siRNA have reached clinical investigation in melanoma.

In addition to heterogeneous genetic changes, there are a range of phenotypic adaptations to BRAF inhibition, indicating that several alternative signaling routes are involved in overcoming BRAF resistance [201]. Because BRAF inhibitor-resistant cells have developed a range of ‘escape routes’, it is likely that multiple different treatment targets will be required to overcome resistance. Therefore, researchers have investigated targeting node points in the activated pathways. Based on the activation/overexpression of transmembrane receptors or RTKs and activation of alternative signaling pathways, some of the novel combinations being tested in vitro include BRAF inhibitors with: TGF-β receptor inhibitors [154], inhibitors of growth factor receptors or RTKs (e.g., gefitinib, sorafenib, dovinitib) [28,53,202,203,204], PI3K/mTOR inhibitors [18,101,205,206,207,208,209,210,211,212,213,214,215,216], bifunctional MAPK/PI3K antagonists [217], anaplastic lymphoma kinase (ALK) inhibitors [35], novel MEK and/or aurora kinase inhibitors [218,219,220,221,222], MAPK activators [223], glucocorticoid receptor antagonists [224], CDK4/6 inhibitors [225], ERK1/2 inhibitors [226,227], CRAF inhibitors [24], pan-RAF inhibitors [228,229,230], heat shock protein (HSP) inhibitors [231,232,233,234,235,236,237], or MCL1 inhibitors [238]. However, few of these combinations have been investigated clinically.

One of the few combinations to be investigated in clinical trials is the combination of a BRAF inhibitor (vemurafenib) with an HSP90 inhibitor (XL888) [239]. This combination was tested in an open-label phase I study in 21 patients with BRAF V600E-mutated metastatic melanoma, but none of the patients had received BRAF inhibitors before, so none had acquired resistance. The objective response rate in the 20 evaluable patients was 75%, with three complete responses and 12 partial responses, and the 1-year OS rate was 60% [239]. The most common grade 3 adverse events were skin toxicities (rash, squamous cell carcinoma, and new primary melanoma), diarrhea, headache, and fatigue [239]. The authors reported that they will be undertaking another clinical trial with the triplet combination of vemurafenib, cobimetinib, and XL888, but note that reduced doses of the BRAF and MEK inhibitors may be needed to limit toxicity with this combination [239].

Epigenetic mechanisms may be targeted by inhibiting key transcription factors, such as WNT5 or STAT3 [134,135,222,240], or by the use of microRNA mimetics [59] or HDAC inhibitors [111,163,241,242,243,244,245]. HDAC inhibitors have also been investigated in combination with a CDK inhibitor with promising in vitro and in vivo activity [233,244,246]. A single-arm, open-label, proof-of-concept study is underway in the Netherlands to investigate the effect of the HDAC inhibitor vorinostat in patients with BRAF V600E-mutated resistant melanoma (NCT02836548) [247]. Patients who progress on treatment with a BRAF inhibitor and/or a combination of a BRAF inhibitor + MEK inhibitor will receive 14 days of treatment with vorinostat, before reinitiating their earlier BRAF/MEK inhibitor treatment. The aim of the study is to see whether vorinostat can purge the resistant clones and re-establish responsiveness to BRAF/MEK inhibitor treatment [247].

Agents targeting the immune system are also being investigated for the treatment of BRAF inhibitor resistance. As described earlier, the use of immune checkpoint inhibitors in combination with a BRAF inhibitor + MEK inhibitor appears to be effective but is associated with a high rate of toxicity [194]. Other potential immune-targeted therapies undergoing preclinical investigation include adoptive T-cell therapy [171,248,249], dendritic cell vaccination [250], and combining BRAF inhibitor treatment with a toll-like receptor 7 agonist (e.g., imiquimod) [160].

A considerable number of preclinical studies are investigating other novel targets for overcoming BRAF inhibitor resistance. These include combining BRAF and/or MEK inhibitors with inhibitors of pre-mRNA splicing (to counteract resistance caused by BRAF splicing) [251], BH3-mimetics [252,253], BCL2 inhibitors [254], mitochondrial-targeted agents [255,256], inhibitors of p90 ribosomal S6 kinases [257,258], pro-caspase activating compounds [259], Rho kinase 1 (ROCK1) inhibitors [260], protein kinase Cδ inhibitors [261], tubulin inhibitors [262], ErbB2 or ErbB3 inhibitors [222,263,264], activators of the liver-X nuclear hormone receptor [265], an antibody conjugate targeting the endothelin B receptor [266], monoclonal antibodies against chondroitin sulfate proteoglycan 4 [267], inhibitors of sterol regulator element binding protein I (SREBP-1) [268], copper chelators [269], polo-like 3 kinase inhibitors (including in models of BRAF + MEK inhibitor resistance) [270,271], anti-nodal antibodies [272], PAK1 inhibitors [273], GLI1/2 inhibitors [274], inhibitors of IQ motif-containing GTPase activating protein 1 (IQGAP1) [275], serotonin agonists [276], CK2 inhibitors [277], p53 activators [278], metformin [279], statins [280], non-steroidal anti-inflammatory drugs [281], mibefradil [282], hydroxychloroquine (an autophagy inhibitor) [83], and A100 (a reactive oxygen species-activated prodrug) [283].

## 4. Future Directions

As described above, considerable research is being undertaken to identify potential new combinations of treatments that may limit or prevent the development of BRAF inhibitor resistance, or overcome resistance once developed. In addition to this, investigations are underway to improve existing therapies, such as employing nanovehicle technology to enhance the safety or targeted delivery of drugs, which may enable the use of higher doses or more potent combinations of existing agents [284,285]. Preclinical studies have investigated the encapsulation of MEK inhibitors in pegylated nanoliposomes for oral administration [284], or the topical administration of BRAF inhibitor-loaded nanovehicles into melanoma lesions using a microneedling technique [285].

The multiplicity of potential targets raises the possibility of using genomic and proteomic data to personalize combination therapy towards the specific pathway that is activated during BRAF inhibitor resistance in individual patients with melanoma [211]. Investigators are developing an MAPK pathway activity score from aggregated gene expression data that could help to determine the best drug combination to use [64], but further validation is needed.

## 5. Conclusions

Given the complexity and heterogeneity of pathways involved in BRAF inhibitor resistance, a ‘one size fits all’ approach to overcoming acquired resistance is unlikely to succeed. However, the plethora of research in this field means that multiple promising leads are being identified and investigated, which bodes well for the development of new treatment approaches for patients with acquired BRAF inhibitor resistance.

## Figures and Tables

**Table 1:** Genetic mutations contributing to acquired BRAF inhibitor resistance (adapted from Tian and Guo, 2020) [13].

Mutation	Mechanism
NRAS mutations [15,31,44,47,49,51,60,61]	Constitutively active RAS mutants enhance BRAF V600E dimerization, reactivate the ERK pathway, and confer resistance to BRAF inhibitor which only block monomeric BRAF V600E
CRAF overexpression, RAF paradox and dimerization of RAF proteins [17,24,36]	BRAF inhibitors can paradoxically activate wild-type BRAF kinase through the induction of dimerization or MAP3K8/COT and CRAF activation, resulting in MEK/ERK phosphorylation and eventually promoting cell proliferation
Secondary BRAF mutations [63,69]	Secondary mutations in V600E (single-nucleotide substitution) or L505H have been detected in patients with BRAF inhibitor resistance. The mutations in V600E increases BRAF kinase activity and causes cross-resistance with MEK inhibitors
BRAF gene amplification and splicing [11,47,50,51,61,62,64,70]	The amplification of the BRAF gene led to significant upregulation of BRAF protein expression, contributing to the reactivation of ERK in the presence of BRAF inhibitors. Alternative splicing can lead to the expression of truncated BRAF proteins that lack the N-terminal RAS-binding domain but retain the kinase domain, which can form homodimers that are resistant to BRAF inhibitor
MEK1/2 mutations [21,44,51,61,62,64]	MEK1/2 mutations could reactivate downstream ERK signaling without the need for BRAF stimulation
Upregulation of membrane receptors, RTKs, or receptor interaction proteins [11,19,20,25,28,29,35,38,39,40,41,46,48,49,52,53,57,58,59,61,66,67]	Overexpression or hyperactivation of membrane receptors/RTKs could promote acquired resistance through the activation of parallel pathways or by direct induction of the RAS pathway; partly mediated by MITF copy gain
Aberrations in the PI3K -AKT pathway [11,14,16,18,23,32,33,34,55,56,68]	PI3K and AKT-activating mutations enhance AKT signaling, which promotes anti-apoptotic signals and upregulates expression of essential proliferative genes, allowing survival signals independently of BRAF
Down-regulation of STAG2 or STAG3 expression [54,68]	Down-regulation of STAG2 or STAG3 expression suppressed CTCF-mediated expression of DUSP6, resulting in the reactivation of ERK
Activation of the YAP/TAZ pathway [14,22,42,45]	The activation of YAP/TAZ pathway after actin remodeling renders resistance to BRAF targeted therapy
Down-regulation of expression of DUSPs [30]	DUSPs are the largest group of phosphatases for dephosphorylating ERK1/2 kinase, DUSPs are considered to be the negative feedback loop of MAPK signaling in response to BRAF-targeted therapy
RAC1 mutation [43,65]	Single-nucleotide variant in RAC1 maintains activation of MAPK pathway via PAK1-mediated co-activation
Somatic mutations in NF1 [27]	Usually a negative regulator of the RAS pathway, inactivation of NF1 expression leads to increased activity in downstream pathways such as PI3K/AKT
Downregulation of expression of RNF125 [37]	Deficiency of RNF125 suppresses ubiquitination and degradation of JAK1, thereby promoting the expression of EGFR that activates downstream ERK signaling and conferring resistance to BRAF-targeted therapy
DBL guanosine exchange factors [26]	Gain-of-function mutations in genes regulating the DBL/RAC1/PAK signaling axis drive resistance to BRAF inhibitors

AKT, protein kinase B; BRAF, v-Raf murine sarcoma viral oncogene homolog B; COT, cancer Osaka thyroid oncogene); CRAF, RAF proto-oncogene serine/threonine-protein kinase; DUSP, dual-specificity phosphatase; EGRF, epidermal growth factor receptor; ERK, extracellular signal-regulated kinase; JAK, Janus kinase; MAPK, mitogen-activated protein kinase; NF1, neurofibromin 1; PAK1, human p21-activated kinase; PI3K, phosphatidylinositol 3-kinase; RAC1, Ras-related C3 botulinum toxin substrate 1; RAF, rapidly accelerated fibrosarcoma; RNF, ring finger protein; RTK, receptor tyrosine kinase; STAG, small T-antigen; TAZ, transcriptional coactivator with PDZ-binding motif; YAP, yes-associated protein.

**Table 2:** Transcription factors and microRNA implicated in BRAF inhibitor resistance.

Transcription Factors	MicroRNA
STAT3 [28,122,131,139]	miR-7 [59]
FLI1 [121]	miR-92a-15p [150,152]
RUNX [123,129]	miR-204-5p [147,149]
YAP [14,22,45]	miR-211-5p [147]
c-MYC [138]	miR-126-3p [146]
Aryl hyodrocarbon receptor [125]	miR-514a [154]
SOX proteins (SOX2, SOX10) [58,126,130,131]	miR-579-3p [148]
β-catenin [120,124,139]	miR-4443 [149]
MITF [46,48,127,130,132]	miR-4488 [149]
MRTF [45]	miR-1246 [151]
JUN [89,97,117,128,136]	miR-200c [153]
ZEB-1 or -2 [66,137]	miR-708-5p [152]
WNT5 [16,120,124,134]	miR-199-5p [149]
NFATc2 [135]	
NRF-1 [133]	
FOXD3 [52]	
E2F1 [140]	
TFEB [81]	

## Supplementary Materials

The following are available online at https://www.mdpi.com/2072-6694/12/10/2801/s1, Supplementary material: Literature search protocols, (1) Genetics; (2) Epigenetics; (3) Immune system; (4) Overcoming resistance.
