# Supplementary material for: Mechanisms of Acquired BRAF Inhibitor Resistance in Melanoma: A Systematic Review
Source: Cancers (Basel). 2020 Sep 29;12(10):2801. doi: 10.3390/cancers12102801 (PMC7600801; doi:10.3390/cancers12102801)
Supplement: Supplementary file 1 [file cancers-12-02801-s001.pdf]

cancers

Supplementary Materials: Mechanisms of Acquired BRAF Inhibitor Resistance in Melanoma: A Systematic Review

Ilaria Proietti, Nevena Skroza, Nicoletta Bernardini, Ersilia Tolino,Veronica Balduzzi, Anna Marchesiello, Simone Michelini, Salvatore Volpe, Alessandra Mambrin, Giorgio Mangino, Giovanna Romeo, Patrizia Maddalena, Catherine Rees and Concetta Potenza

Literature search protocols.

(1)Genetics.

Database(s): **Ovid MEDLINE(R) and Epub Ahead of Print, In-Process & Other Non-Indexed Citations and Daily** 1946 to May 18, 2020
Search Strategy:

#	Searches	Results	Annotations
1	exp Melanoma/	94173	
2	melanoma*.mp.	133836	
3	1 or 2	134023	Disease set
4	((BRAF* or "B-RAF*" or "*V600" or "*V600E*" or "V600K*" or "V600R*") and "inhibitor resistance").ti,ab,kw.	148	
5	((BRAF* or b-raf*) adj3 resistance*).ti,ab,kw.	629	
6	4 or 5	668	BRAFi
7	exp DNA/ or exp Genetics/ or exp Sequence Analysis, DNA/	1103152	
8	("deoxyribonucleic acid" or dna? or microRNA? or miRNA? or gene? or genom* or genotyp* or chromosome* or sequenc* or "polymerase chain reaction" or PCR).ti,ab,kw.	3743363	
9	(("deoxyribonucleic acid" or dna? or microRNA? or miRNA? or gene? or genetic* or genom* or genotyp* or chromosome* or cell? or extracell* or extra-cell* or molecular* or metabolic* or BRAF* or B-RAF* or MEK* or KRAS* or NRAS* or MAPK*) adj5 (resist* or mutat* or alterat* or amplificat* or deletion* or adapt* or switch* or inhibit* or plastic* or rewir* or differentiat* or de-differentiat* or proliferat*)).ti,ab,kw.	1524726	
10	7 or 8 or 9	4622291	Genetics set
11	3 and 6 and 10	524	
12	(case reports or clinical conference or congress or news or newspaper article or "review" or conference or meeting or abstract or presentation? or symposium?).pt.	4895193	
13	11 not 12	418	
14	limit 13 to yr="2010 -Current"	414	
15	limit 14 to "humans only (removes records about animals)"	408	
16	remove duplicates from 15	405	Download set

(2) Epigenetics.

Database(s): **Ovid MEDLINE(R) and Epub Ahead of Print, In-Process & Other Non-Indexed Citations and Daily** 1946 to May 18, 2020
Search Strategy:

#	Searches	Results	Annotations
1	exp Melanoma/	94173	
2	melanoma*.mp.	133836	
3	1 or 2	134023	Disease set
4	((BRAF* or "B-RAF*" or "*V600" or "*V600E*" or "V600K*" or "V600R*") and "inhibitor resistance").ti,ab,kw.	148	
5	((BRAF* or b-raf*) adj3 resistance*).ti,ab,kw.	629	
6	4 or 5	668	BRAFi
7	exp Epigenomics/ or exp Epigenesis, Genetic/	127602	
8	(epigenetic* or epi-genetic* or epigenomic* or epi-genomic* or epigenesis or epi-genesis or epimutation or epi-mutation).mp.	92415	
9	DNA Methylation/ or exp transcription, genetic/ or exp transcriptome/ or exp Epithelial-Mesenchymal Transition/ or transcriptom*.ti,ab,kw. or ((methylation or histone or phenotype*) adj3 (modif* or change*)).ti,ab,kw.	334967	
10	7 or 8 or 9	463133	Epigenetic or transcriptomic changes set
11	3 and 6 and 10	53	
12	(case reports or clinical conference or congress or news or newspaper article or "review" or conference or meeting or abstract or presentation? or symposium?).pt.	4895193	
13	11 not 12	47	
14	limit 13 to yr="2010 -Current"	47	
15	limit 14 to "humans only (removes records about animals)"	46	
16	remove duplicates from 15	46	Download set

(3) Immune system.

Database(s): **Ovid MEDLINE(R) and Epub Ahead of Print, In-Process & Other Non-Indexed Citations and Daily** 1946 to May 18, 2020
Search Strategy:

#	Searches	Results	Annotations
1	exp Melanoma/	94173	
2	melanoma*.mp.	133836	
3	1 or 2	134023	Disease set
4	((BRAF* or "B-RAF*" or "*V600" or "*V600E*" or "V600K*" or "V600R*") and "inhibitor resistance").ti,ab,kw.	148	
5	((BRAF* or b-raf*) adj3 resistance*).ti,ab,kw.	629	
6	4 or 5	668	BRAFi
7	exp Immune System/	1144069	
8	(immune or immunity or "cell-mediated" or antibod* or antigen* or protect* or detect* or defense* or complement).ti,ab,kw.	4458764	
9	7 or 8	5074871	immune system set
10	3 and 6 and 9	117	
11	(case reports or clinical conference or congress or news or newspaper article or "review" or conference or meeting or abstract or presentation? or symposium?).pt.	4895193	
12	10 not 11	87	
13	limit 12 to yr="2010 -Current"	85	
14	limit 13 to "humans only (removes records about animals)"	83	
15	remove duplicates from 14	82	Download set

(4) Overcoming resistance.

Database(s): **Ovid MEDLINE(R) and Epub Ahead of Print, In-Process & Other Non-Indexed Citations and Daily** 1946 to May 18, 2020
Search Strategy:

#	Searches	Results	Annotations
1	exp Melanoma/	94173	
2	melanoma*.mp.	133836	
3	1 or 2	134023	Disease set
4	((BRAF* or "B-RAF*" or "*V600" or "*V600E*" or "V600K*" or "V600R*") and "inhibitor resistance").ti,ab,kw.	148	
5	((BRAF* or b-raf*) adj3 resistance*).ti,ab,kw.	629	
6	((overcom* or over-com*) adj1 resist*).ti,ab,kw.	2876	
7	("Mitogen-Activated Protein Kinase*" or MEK1* or MEK2*).ti,ab,kw.	52162	
8	((Vemurafenib* or Zelboraf* or Dabrafenib* or Taffiner* or Tafinlar* or Tafinrar* or Encorafenib* or Braftovi*) and ("MEK inhibit*" or MAPKi or Cobimetinib* or Cotellic* or Trametinib* or Mecinist* or Mekinist* or Mekinisuto* or Binimetinib* or Balimek* or MEK-162 or MEK162 or Mektovi*)).mp.	1007	
9	(4 or 5) and (6 or 7)	155	
10	3 and (8 or 9)	892	Melanoma disease set and ways to overcome resistance to BRAF inhibitors
11	(case reports or clinical conference or congress or news or newspaper article or "review" or conference or meeting or abstract or presentation? or symposium?).pt.	4895193	
12	10 not 11	510	
13	limit 12 to yr="2010 -Current"	509	
14	limit 13 to "humans only (removes records about animals)"	506	
15	remove duplicates from 14	499	Download set

© 2020 by the authors. Licensee MDPI, Basel, Switzerland. This article is an open access article distributed under the terms and conditions of the Creative Commons Attribution (CC BY) license (http://creativecommons.org/licenses/by/4.0/).
